# Beliefs about HIV cure: A qualitative study of people living with HIV in Soweto, South Africa

**DOI:** 10.4102/sajhivmed.v26i1.1644

**Published:** 2025-01-30

**Authors:** Fatima Laher, Naledi Mahlangu, Mbalenhle Sibiya

**Affiliations:** 1 Perinatal HIV Research Unit, Faculty of Health Sciences, University of the Witwatersrand, Johannesburg, South Africa

**Keywords:** HIV, cure, qualitative, perception, belief, traditional, conventional, medication

## Abstract

**Background:**

Rare cases of HIV cure exist. Clinical trials of HIV cure are also underway. However, little is documented about how potential cures are perceived by African people living with HIV, although they are key stakeholders.

**Objectives:**

We explored knowledge, beliefs, and experiences about HIV cure in Soweto, South Africa.

**Method:**

We conducted qualitative research with five stratified focus groups (*N* = 49). Consenting adults living with HIV were eligible. Facilitators asked participants about their knowledge of HIV cure, experience of purported cures, and beliefs about cure possibilities. Transcripts from audio recordings were thematically analysed.

**Results:**

Participants had knowledge of the concept of cure as eradication, not remission. Three main themes emerged about possible HIV cures. Firstly, hope and scepticism: people feared unequal access to technologies. Secondly, cultural and conventional approaches: there were beliefs in traditional healers, scepticism towards culturally purported cures (e.g. *imbiza* herbal tonic), and a desire for medical cures to obviate pill burdens. Thirdly, anticipated socio-behavioural effects: beliefs existed that cures might improve happiness, reduce emotional burdens of disclosure, facilitate HIV-free generations, increase risk behaviours, and reduce health checks, but not change societal attitudes to HIV.

**Conclusion:**

In Soweto, South Africa, people living with HIV hope for medical technologies – such as cure and long-acting treatments – to relieve the biopsychosocial burdens of chronic treatment. Despite treatment knowledge, some people try culturally purported cures for HIV. In HIV cure trials, consent language should avoid ‘cure’ when remission is meant. Care should address pill burden, and counselling should address sex, substances, exercise, and nutrition.

**What this study adds:** Our findings advocate for expansion of HIV cure research in South Africa to address chronic pill burden. Our findings elucidate the biopsychosocial context of emerging HIV cure research in Soweto, South Africa, yielding counselling recommendations.

## Introduction

Currently, HIV cure is defined as viral eradication (‘sterilising’) or treatment-free remission (‘functional’).^1^ There are exceptional case studies of people documented as cured from HIV after stem cell transplants,^2,3,4,5,6,7^ post-treatment control,^8,9,10^ and spontaneous control.^11^ Research to find safe and scalable HIV cures is gaining momentum, in part because of long-term challenges to living with HIV, including lifelong antiretroviral therapy (ART) adherence and stigma.^12^

Most HIV infections globally are in Africa and among women, populations that should be stakeholders in the search for a cure.^13^ Disconcertingly, an analysis of 73 HIV cure-related clinical trials and observational studies in 2018 revealed under-representation of African people and women.^14^ The opinion of some researchers is that cheap, simple, brief and safe HIV cures would be appropriate to develop for Africa.^1^ However, the intervention itself should not be the only consideration: psychosocial contexts also embed health intervention research and implementation. Social norms, individual experiences, community experiences and individual reasoning may shape beliefs; studies have shown that beliefs vary among populations and may influence health. Examples include trust in healthcare systems and vaccination differing by country,^15^ perceptions of the effectiveness of different administration routes differing by region,^16^ and the influence of religious and spiritual beliefs on the physical wellbeing of people with cancer.^17^

How African communities view HIV cure possibilities within the African context of cultural beliefs and societal attitudes is largely undocumented. Yet such beliefs are relevant, because they plausibly influence adherence to treatment, the deleterious use of purported unsubstantiated cures, willingness to participate in HIV cure research, and the acceptability of potential cures. A study in Tanzania found that the cultural and religious belief in a cure for HIV through prayer was not associated with treatment refusal.^18^ In Ghana, people living with HIV were risk averse to hypothetical HIV cure research: although the authors did not investigate reasons, they suggested a link with certain beliefs such as perceptions of harm, and low trust in the medical establishment.^19^

Murdock’s ill-health theoretical model informed our exploration of the beliefs and experiences around purported cures.^20^ In Murdock’s framework, there are beliefs about the natural and supernatural causes of disease, which may influence beliefs about cure. In South Africa, there is a predominating natural belief, consistent with biomedicine, viewing viral infection as the cause of HIV. However, there co-exists a traditional African supernatural belief paradigm viewing the cause of HIV as mystical (e.g. ancestral retribution), animistic (e.g. loss of soul), and magical (e.g. witchcraft). Under the supernatural paradigm, the belief of traditional healing is that it could cure some forms of HIV.^21^

We also sought to understand what people living with HIV believed would be potential consequences of HIV cure for themselves and communities. When viewed within Merton’s theory of unanticipated consequences of purposive social action, the research and implementation of an HIV cure could have multiple effects: desired, unintended negative, negative related to desired, and unintended positive consequences.^22^ Theoretical examples of unintended negative consequences could be job loss within the HIV sector, and loss of identity amongst people living with HIV.^23^

Responding to calls for diversity, we aimed to study what people living with HIV across the gender spectrum know and believe about HIV cure possibilities in South Africa, the country with the highest number of people living with HIV. As HIV cure trials emerge in South Africa, it is useful to understand how to tailor these trials within the framework of local knowledge, beliefs, and experiences. Here we used a qualitative approach to uncover nuanced insights into knowledge, experiences, and beliefs about HIV cure possibilities.

## Research methods and design

### Study design

This cross-sectional qualitative study was an initial component of a larger ongoing mixed-methods study about acceptability of HIV cure possibilities.

### Setting

We conducted the study during 2024 at the Perinatal HIV Research Unit in Soweto, where the estimated population size is 3 million people within 63 km^2^.^24^ Soweto is a peri-urban township in South Africa’s Gauteng province, where estimated HIV prevalence is 17.6% of the adult population aged 15 years – 49 years.^25^

### Study population and sampling strategy

Eligible people were aged 18 years and older, who self-reported that they were living with HIV and had never experienced HIV cure, which we defined in the study as undetectable viral load after being off treatment for at least 6 months. We excluded individuals with any condition deemed to represent a challenge to safe and meaningful group participation, for example impaired hearing, inability to speak, impaired cognition, acute infectious disease transmissible within a group setting, and anxiety or unwillingness to discuss HIV. To mitigate HIV stigma, we recruited participants for the focus groups through the snowball sampling strategy, in which people living with HIV referred other potential participants. To minimise power dynamics, we stratified the focus groups by gender identity and age. To enhance the possibility of open discussion, we limited each focus group to 10 participants.^26^ We planned five focus groups because methodological studies had determined that five focus groups permit saturation for thematic analysis.^27^

### Data collection

Two trained multilingual facilitators conducted each focus group for 60 min – 95 min in commonly spoken local languages (English, isiZulu, Sesotho, isiXhosa and Afrikaans). The facilitators used a semi-structured guide that had been refined during pilot testing toward non-leading, open-ended questions, and probes about baseline knowledge, experiences, and beliefs about HIV cure (Table 1). We did not train participants on the discussion topic beforehand, because we sought to document their pre-existing ideas. At the end of the focus group discussion, facilitators reminded participants that HIV cures are unavailable, so it is best to continue ART. Facilitators audio-recorded the discussion for verbatim transcription and translation to English. We checked transcripts for quality.

**TABLE 1:** Semi-structured guide.

Focus group guide questions about knowledge, experiences, and beliefs about HIV cure
1. What do you understand by the words ‘HIV cure’?
2. What do you know about research to get an HIV cure?
3. What have you tried, or heard that other people in your community tried, to cure themselves of HIV that your doctors and/or nurses do not discuss?
4. How would a cure affect you or the people you know who are living with HIV?
5. What are your and your community’s beliefs about HIV cure?

### Data analysis

We performed thematic analysis^28^ manually. A trained analyst read transcripts to become familiar with the data, and then assigned codes to excerpts of text. Codes were not pre-determined, but rather emerged from the data (Table 2). The analyst met with two of the researchers (who were also facilitators) to refine the codes and themes through discussions and re-readings of the transcripts, until they agreed on a final list. We selected quotations to exemplify the themes; we preserved the anonymity of the speaker by crediting them to their focus group, and not to individuals.

**TABLE 2:** Coding tree of codes and themes that emerged from the data.

Themes	Codes
Hope and scepticism about HIV cure possibilities	Hope for a cure
	Scepticism about a cure
	Cure difficulties
	Distrust in authorities
	Unequal access
Cultural and conventional approaches to cure	Beliefs about disease cause and treatment
	Traditional medicine
	Cure myths leading to sickness and death
	Acceptance of HIV status and treatment
	Willingness to try new conventional medicines
	Experiences with daily HIV treatment
Anticipated effects of HIV cure on behaviour and society	Happiness
	Sexual pleasure
	No change
	Risky behaviour
	Pregnancy and future HIV-free generations
	Regular health check-ups
	HIV disclosure

### Ethical considerations

Ethical clearance to conduct this study was obtained by the University of the Witwatersrand Human Research Ethics Committee (Medical) approval number: M240131 M240426-A-0001. All participants provided written voluntary informed consent. Data are reported without identifiers to maintain confidentiality.

## Results

### Demographics

Forty-nine people participated in one of five focus groups: *n* = 9 female participants, 18–40 years old; *n* = 10 female participants, 41 years and older; *n* = 10 male participants, 18–40 years old; *n* = 10 male participants, 41 years and older; and *n* = 10 non-binary persons, 18 years and older.

### Knowledge about HIV cure

In all focus groups, participants said they did not know much about cures or related research, and would want to learn more. However, they had knowledge, which was comforting to them, that although antiretroviral pills cannot cure HIV, they suppress HIV lifelong:

‘If we take medication there is hope and without medication there is no hope.’ (Male, 41 years or older, living with HIV)

Participants’ understanding of the word ‘cure’ aligned with scientific meanings of complete eradication:

‘[*There*] won’t be a virus in the blood and in the body.’ (Female, 18-40 years old, living with HIV)

‘We will no longer take treatment, right? Cure means it [*HIV*] is no longer there.’ (Female, 41 years or older, living with HIV)

‘[*C*]ure is a permanent solution …’ (Male, 41 years or older, living with HIV)

‘A cure is something that will get rid of it totally out of our system. Then we become clean.’ (Male, 18–40 years old, living with HIV)

‘… HIV cure is eliminating it from your system completely.’ (Non-binary gender, 18 years or older, living with HIV)

### Theme 1. Hope and scepticism about HIV cure possibilities

Hope and desire for a cure co-existed with scepticism about the feasibility of its discovery because of past research disappointments, the complexity of the virus, and individual variation in response. During some focus groups, there was a belief that government and pharmaceutical companies are hiding a cure from the public for a profit motive:

‘I agree with [*another focus group participant*]. The government is benefiting a lot with the treatment of HIV, so if they find a cure … they will lose millions of money, so I don’t think the cure will come.’ (Female, 18–40 years old, living with HIV)

‘It’s not about that the fact that they don’t have it [*the cure*]: they have it, but they are holding it back. As far as the child in my womb, it is protected and I’m gonna give birth to the child, the child will come negative. So, and also even if a woman is raped, also they have got that tablet they give that woman not to be affected. So that means somewhere somehow, I see that the government is playing with our minds.’ (Female, 41 years or older, living with HIV)

There was a sub-theme of unequal cure access in some focus groups. Female participants 41 years and older believed that those who had been living longest with HIV should receive priority to access a hypothetical cure. Other participants believed that disparities would affect cure access:

‘The government will sell the cure for the people who have money. If you have money, you can cure yourself, but when you don’t have money, you take pills. Because the company of ARVs [*antiretrovirals*] they won’t allow it to lose billions of money just because of a cure that appears recently in the 21st century, so I don’t think there will be cure for each and every one of us.’ (Female, 18–40 years old, living with HIV)

‘In South Africa, we do have that cure. It was released so people can be okay, but they were gatekeeping us.’ (Male, 18–40 years old, living with HIV)

### Theme 2. Cultural and conventional approaches to cure

There was a belief that witchcraft causes some diseases curable only by traditional healers who practise ancestral healing. Some participants believed that traditional healers who had found effective cures for HIV had been murdered:

‘Pills are like a serious business, like a very serious business: these people are making a lot of money. So, traditional healers sometimes they are sidelined because they have already found a cure, so they decide to kill them …’ (Male, 18–40 years old, living with HIV)

Multiple cultural approaches to HIV cure were named, including *imbiza* [traditional herbal tonics made from plant extracts], *Uvukahlale* [traditional herbal medicine], ‘booster’ medications, ‘drinking the water from beetroot and eating it raw’, teas from churches (faith healing), initiation to become a traditional healer, anal douching, emetics, and enemas. Participants discussed sex with a virgin, a gay man, a child, or a person with albinism as outdated harmful myths that persist in some places.

*Imbiza* was the dominant cultural approach to HIV cure, and there were varying experiences of its effectiveness. Some participants spoke of their experience of *imbiza* helping manage HIV-related infections, while others stated experiences that *imbiza* did not eliminate HIV but may cause morbidity and mortality. Participants also believed there are potential drug interactions if taking *imbiza* and conventional medicines simultaneously:

‘People have had the mindset that HIV is in the blood, so that’s why they will come and say that there is imbiza to clean the blood.’ (Female, 41 years or older, living with HIV)

‘I would drink three spoons [*of imbiza*] … When I went to the clinic, I saw that there, in a case where it [*viral load*] was supposed to go down, it rather went up.’ (Female, 18–40 years old, living with HIV)

‘I went to a certain traditional healer because a friend of mine convinced me to. I didn’t even last. I ended up sleeping here at [*a hospital*]. For 2 years after I returned from that traditional healer who made me drink imbiza, my feet swelled, my face swelled, my skin was peeling everywhere … I was told that that guy [*the friend*] had died from taking imbiza we were using.’ (Male, 41 years or older, living with HIV)

‘[*Y*]ou educate them to drink imbiza but also take their treatment, but not both at the same time so that the pills can work and imbiza can also work the way it is supposed to …’ (Female, 18–40 years old, living with HIV)

Generally, participants spoke about unconventional cures with attitudes of caution and scepticism, believing that people who tried these approaches were in denial of their HIV status, became sicker or demised:

‘[*T*]hey respond, “They say I have an ancestral calling”, ”They say I was bewitched” – only to find that, where is the problem? The problem [*HIV*] is in the blood.’ (Female, 41 years or older, living with HIV)

Some, particularly female participants between 18 and 40 years old, questioned the need for a cure. They believed that HIV was more manageable than other comorbidities, and part of their identity of belonging to the community of people living with HIV. Knowing what to expect when on the daily routine HIV treatment was familiar and comforting to these participants’ sense of wellbeing. They believed that seeking HIV cures would be unnecessary if a person accepted their HIV status and used conventional medically prescribed daily antiretroviral treatments:

‘For me it [*a cure*] wouldn’t make a difference because I have already accepted my life as it is.’ (Female, 18–40 years old, living with HIV)

‘I think the pills work better than the cure that will come, because other people consider it [*HIV*] to be like flu. Like as long as there is a pill, they don’t mind having it [*HIV*].’ (Female, 18–40 years old, living with HIV)

Others stated strong positive attitudes to the reduction or elimination of daily treatment through conventional medicine approaches, such as novel long-acting injectable treatments, and participation in medical cure research. These attitudes were influenced by having experienced pill fatigue (particularly evident in women over 41 years old who expressed frustration about long-term treatment and the lack of a cure), the stigma of being seen taking medication, side effects of oral antiretrovirals, and negative experiences of discomfort and stigma in healthcare facilities:

‘I have drunk pills so much. I have drunk them! I would be happy if they were to say it’s here now and they are giving us the [*cure*] injection now! Even right now, I would take the injection, I would inject it now now I would inject it.’ (Female, 41 years or older, living with HIV)

‘[*W*]hat I think about this cure research that I am not sure about, is how this cure that’s coming is going to affect us as people? Because we’ve been on pills and they have different effects on all of us: some of them get diarrhoea, some of them get burned on their faces, some can’t use their feet to walk anymore, and all these other things. So, with this cure research, maybe they need to get the injection.’ (Male, 41 years or older, living with HIV)

‘It [*ARV pills*] has depression. It makes you crazy. It’s disgusting. It makes you vomit and it’s irritating. And when you’re done drinking it, you feel like murdering. You want to burn something.’ (Non-binary gender, 18 years or older, living with HIV)

‘It’s [*ARV pills*] big. It sits here [*throat*] as well.’ (Female, 41 years or older, living with HIV)

‘It’s been 15 years since I started taking treatment. And then the government must help us with this HIV that we also take a break from taking pills. Taking pills is not nice. At times you forget, then you wake up in the middle of the night and realise that you forgot to drink your pills. Sometime it’s tiresome … your mind gets tired just by looking at it, that is: this is making me tired, how long have I been drinking it, and how long for I have to keep drinking it, what is it affecting in my body, is it these pills making me gain weight or this pill making me more sick? … Here now Covid [*COVID-19*] arrived, and we were able to get injections, and they said that “Covid has decreased, Covid has come to an end”. We also want that time to arrive when HIV decreases and comes to an end. We are tired and we have had enough.’ (Female, 41 years or older, living with HIV)

### Theme 3. Anticipated effects of HIV cure on behaviour and society

Participants said that receiving a cure might have emotional effects of happiness and less worry:

‘I would bring myself to be the first volunteer to get that [*cure*] injection, because I need to live a more anxiety-free life. Because on a serious note I could tell you: each and every one that is taking HIV treatment, there are just days where we just go without taking it [*pills*].’ (Non-binary gender, 18 years or older, living with HIV)

There were beliefs that a cure might change behaviour. Some believed that people might increase unprotected sex, resulting in greater sexual pleasure but unwanted consequences of sexually transmitted infections and teenage pregnancies. However, in the group with female participants 41 years and older, there was a belief that being cured might intensify the burden of using condoms to prevent re-infection. Also, there was a belief that a cure might lead to complacency about health checks in the absence of routine HIV monitoring, and other lifestyle-related health behaviours:

‘The cure would affect people, because we don’t have HIV only as a sickness: we have STDs [*sexually transmitted diseases*]. Because people are going to stop using protection condoms. What will happen is they will have herpes STDs, STIs [*sexually transmitted infections*], such things … They be like, “No more condom. Skin on skin.”’ (Non-binary gender, 18 years or older, living with HIV)

‘We would be really happy. But now the thing is, you’ll be doing things that you’ve never done before: now you have to start using condoms.’ (Female, 41 years or older, living with HIV)

‘So, once we find a cure we will now start to stay relaxed, not going to the clinics. You don’t know what illness you have and what is happening, even when you are sick, you’ll say, “No, it will go away”. So now as we are positive, we are able to go to the clinics and have bloods drawn from us and check if your blood is still okay, is there any other infection that you have in your body.’ (Female, 18–40 years old, living with HIV)

‘They won’t eat healthy anymore … People are going to abuse alcohol even more, they won’t care because, whatever happens, there is a cure, right? Even smoking. People will not exercise anymore.’ (Male, 41 years or older, living with HIV)

While some believed a cure may not meaningfully shift societal attitudes about HIV nor change the daily life of a person who was in a state of acceptance about their HIV status, others believed it would:

‘Psychologically, it [*the cure*] would help me, cause this thing of fetching medication is a bore, feels like a job.’ (Male, 41 years or older, living with HIV)

During some focus groups, the idea emerged that a cure could help prevent vertical transmission, help future generations remain HIV free, and reduce the burden to disclose HIV status:

‘[*W*]hen you find out your status, not everyone is comfortable to disclose to their partners and family and stuff … so it also helps if there is a cure. You know you are safe: you don’t have to go around disclosing your status with every guy that you meet and stuff, so it saves as well.’ (Female, 18–40 years old, living with HIV)

## Discussion

Our findings contextualise pre-existing ideas that some people living with HIV in Soweto, South Africa, have about HIV cure and research for a cure. Generally, Soweto participants had relevant knowledge about ART, but little knowledge about cure research, had hope and scepticism about HIV cure possibilities, were cautious about cultural approaches to HIV cure, desired conventional approaches to HIV cure, and anticipated both positive and negative effects of HIV cure on behaviour and society.

Although some – mainly younger female participants – expressed contentedness with their HIV status as a part of adjustment and identity formation, the prevailing experience was feeling fatigued from the challenges of treating HIV chronically. Resultantly, many were enthusiastic about what they believed would be the benefits of potentially being cured. Our findings suggest that some South Africans living with HIV might consider strategies toward remission to relieve their issues with daily pill taking, even temporarily. However, while participants knew that long-acting injectable antiretrovirals are on the horizon, they mostly understood the word ‘cure’ to be eradication, an outcome that they generally desired. ‘Cure’ is thus a word loaded with the hope of achieving permanent removal of HIV from the body – not temporary remission – so we caution trialists to communicate clearly in consent and protocol documents to avoid misconceptions. Cure research literacy efforts may be appropriate to engage communities because we found that patients are generally unfamiliar with cure research.

A smaller study in another province in South Africa also found that people living with HIV, community stakeholders and research staff had little knowledge of HIV cure research. Unlike our study, there was much cure pessimism.^29^ This is possibly because that study was older, and more verified cure cases had been publicised by the time we conducted our study.

In other regions, research on perceptions of an HIV cure also found HIV cure optimism co-existing with scepticism. Unlike the study of Chinese participants who were disinterested in cures because they stated they had adjusted to life with HIV,^30^ many of our South African participants expressed frustration with chronic HIV and their desire for cure. A few studies have found that HIV cure is a concept that carries social meaning, accentuating the need to consider context-specific social implications in research and implementation. For example, people living with HIV and key populations in the Netherlands perceived that a cure might reduce transmission risk and alleviate HIV stigma.^31^ However, people who injected drugs and men living with HIV who have sex with men in China perceived that a cure could reduce, exacerbate, or not change HIV discrimination.^32,33^

Our study contributes documentation of the non-Western methods – mainly herbal – attempted by South Africans living with HIV to effect a cure from HIV, and their experience of not only non-efficacy but also harm. For any of the described methods, evidence for cure does not exist, indicating that, while there is a gap in conventional medicine for a cure, cultural and social influences may persuade people living with HIV to try different strategies purported to cure. Previously, it was documented that about a third of South Africans living with HIV used herbal remedies, including *imbiza*, as part of HIV management, including for analgesia, immune restoration, and to stop diarrhoea.^34^

Our findings suggest some recommendations, underpinned by a biopsychosocial framework, to scientists and funders pursuing cure trials in South Africa. First, we consider our finding that people hope for a cure in part because of pill burden: the fatigue of taking large pills consistently, the stigma that people living with HIV may face with the visible use of pills, and drug side effects. People living with HIV value pills as crucial to their health, but still experienced challenges that they tried to resolve by suboptimal adherence or culturally purported cures. They also described pill burden as a motivation to participate in cure research. Because trial participation does not assure cure, researchers and participants should have reasonable plans to navigate pill-taking distress, for example offering long-acting alternatives to daily pills such as injectable treatments, identifying and managing depression and anxiety, and enlisting adequate social support for adherence. Trials are also investigating the risks and benefits of reducing the number of days that long-term suppressed patients take pills.^35^ Antiretrovirals are often hailed as a victory of transforming HIV into a chronic condition.^36^ However, this should not numb us to the frustrations experienced by many who have lived with HIV for much of their lives, as documented in our study and others.^37^

Second, we elaborate on our findings about distrust. It would be facile to dismiss these as conspiracy theories. Instead, we interpret this as being rooted in fears of unequal access, and awareness of motives that do not always serve African communities. Despite participation in clinical trials, African communities reliant on pharmaceutical imports have often been marginalised by unequal and delayed access to medical technologies, relying on pharmaceutical imports. This is not only historical, but also occurred in recent memory with COVID-19 vaccines and treatments, and HIV long-acting drugs.^38^ We recommend that the informed consent process is transparent about post-trial cure access to build trust, enhance research participation, ensure ethical practice, and manage expectations.

Third, our findings have implications for procedures that may be of importance to participants in cure trials, for example health monitoring, and risk reduction counselling that extends beyond sex to broader lifestyle behaviours of alcohol, tobacco, activity, and nutrition.

A key strength of our study is that the gender and age diversity of our participants represents a wide range of views, unlike previous studies that included specific populations. The main limitation of this study is that its setting at a peri-urban hospital probably resulted in under-representation of people who may have even more challenges with pill treatments and may therefore be more likely to desire a cure, for example people living in rural areas and people not taking ART. To avoid excluding the latter, we did not recruit from a hospital database, but rather by snowballing in community networks.

## Conclusion

In Soweto, South Africa, people living with HIV hope for medical technologies such as cure and long-acting treatments that will relieve biopsychosocial burdens of chronic HIV. Integrated strategies are needed to address pill fatigue, stigma, and the use of ineffective and sometimes harmful non-conventional purported cures.
